# Optical coherence tomography reveals retinal structural abnormalities in α-synucleinopathies: insights from the Padua-CESNE cohort

**DOI:** 10.1007/s00702-025-02918-y

**Published:** 2025-04-15

**Authors:** M. Campagnolo, M. Puthenparampil, A. Emmi, L. Weis, E. Basili, V. Mauceri, A. Miscioscia, M. Carecchio, A. Guerra, V. Misenti, C. Fogliano, P. Gallo, A. Antonini

**Affiliations:** 1 Parkinson and Movement Disorders Unit, Centre for Rare Neurological Diseases (ERN-RND), Department of Neuroscience, University of Padova, Via Giustiniani 3, 35121 Padua, Italy; 2 Center for Neurodegenerative Disease Research (Centro Studi per la Neurodegenerazione CESNE), University of Padova, Padua, Italy; 3 Multiple Sclerosis Centre, Department of Neuroscience, University of Padova, Padua, Italy; 4 Institute of Human Anatomy, Department of Neuroscience, University of Padova, Padua, Italy; 5 IRCCS San Camillo Hospital, Venice, Italy

**Keywords:** Parkinson’s disease, Multiple system atrophy, Optical coherence tomography, α-synuclein, Biomarkers

## Abstract

The complexity of α-synucleinopathies, namely Parkinson’s disease (PD) and multiple system atrophy (MSA), calls for the adoption a multimodal approach integrating biological, morphological, and functional data. *Phosphorylated* α-synuclein (α-syn) detection in bodily fluids and tissues such as the skin helps provide biological characterization of the disease, but specific and accessible biomarkers are not available yet. The aim of this study was to define the role of Optical Coherence Tomography (OCT, a minimally invasive retinal imaging technique) patterns as possible biomarkers in the early stages of α-synucleinopathies, also supporting the differential diagnosis. Thirty-five (23 PD, 12 MSA), clinically, biologically and genetically characterized patients included in the PADUA-CESNE (Centro Studi per la Neurodegenerazione) cohort underwent OCT. A significant atrophy in the inferior, superior and temporal regions of the Retinal Nerve Fiber Layer (RNFL) and in the inner nuclear layer (INL) were observed in PD compared to controls, differently from MSA. Hyperreflective foci (HRF) counts were elevated across all retinal layers in all patients with PD exhibiting significantly higher numbers, suggesting microglial activation and greater retinal damage. Further research regarding OCT patterns in PD and MSA may consolidate the role of specific features, such as INL abnormalities and different HRF counts, in supporting the diagnosis and differential diagnosis in α-synucleinopathies. In light of the availability of potentially disease-modifying therapies, studies should focus on newly diagnosed patients, also undergoing thorough clinical, biological and genetic characterization.

**Supplementary Information:**

The online version contains supplementary material available at 10.1007/s00702-025-02918-y.

## Introduction

The intricate nature of α-synucleinopathies, such as Parkinson’s disease (PD) and multiple system atrophy (MSA), and the potential involvement of multiple organs and tissues in their pathological processes are driving the adoption of a diagnostic multimodal approach. This approach integrates biological, morphological, and functional data to achieve an early disease detection before symptoms become clinically manifest (Outeiro et al. 2023; Chopra et al. 2024). Early identification is crucial for effective symptomatic management and becomes even more relevant with the emergence of potentially disease-modifying therapies (McCann et al. 2014; Singer 2022). Biological characterization has become increasingly included in the diagnostic work-up of α-synucleinopathies, thanks to the possibility of detecting phosphorylated α-synuclein (α-syn) in bodily fluids such as cerebrospinal fluid (CSF) and blood, as well as in peripheral tissues like the skin and gastrointestinal tract^5^. In recent years skin sample analyses and particularly immunohistochemistry (IHC), have yielded promising outcomes in providing biological support to the clinical diagnosis by detecting and quantifying pathological α-syn (Magalhães and Lashuel 2022; Peng et al. 2023). Early involvement of cutaneous nerve fibers in the disease process was observed, underscoring the potential of skin biopsy as a valuable tool for identifying PD in its early and prodromal stages^7^. Additionally, distinct patterns of α-syn distribution and nerve fiber densities have been observed across various clinical entities. The identification of a pathological signature specific to PD and multiple system atrophy (MSA) might support clinical and radiological diagnostic criteria and facilitate the identification of subgroups based on differing clinical and molecular features, potentially aiding in the definition of at-risk cohorts (Donadio et al. 2020; Gibbons et al. 2023, 2024).

However, the quest for accurate, easily accessible and minimally invasive biomarkers remains an unmet need.

Optical Coherence Tomography (OCT) is a minimally invasive imaging technique that combines high-resolution in vivo cross-sectional retinal imaging capability with quantitative anatomical data and has shown promising results in multiple sclerosis (MS) and many other neurological disorders (Mirmosayyeb et al. 2023; Ahn et al. 2016). Notably, consistent alterations in retinal and vascular structures have been observed (London et al. 2013). In PD, OCT's utility stems from dopamine's pivotal role in supporting retinal function, alongside the identification of phosphorylated α-synuclein aggregates in the inner retina (Lee et al. 2022). Additionally, studies have highlighted thinner peri-papillary retinal nerve fiber (pRNFL) and inner retinal (IRL) layers in PD patients compared to healthy individuals (Lee et al. 2022; Zhou et al. 2021). Furthermore, these features may reflect disease progression and exhibit distinct patterns correlating with clinical stage and severity, while their role in the differential diagnosis among α-synucleinopathies is still uncertain (Lee et al. 2022).

The aim of this study was to describe patterns of retinal structure abnormalities in patients with PD and MSA from our PADUA-CESNE (Centro Studi per la Neurodegenerazione) cohort, defining the role of OCT as biomarker in the early stages and supporting differential diagnosis.

## Methods

All patients are part of the PADUA-CESNE dataset, undergoing clinical, biological, and genetic characterization at our institution (Bonato et al. 2024; Carrer et al. 2024).

PD diagnosis was made according to the 2015 International Parkinson and Movement Disorder Society (MDS) criteria (Postuma et al. 2015), considering patients with disease duration < 5 yrs (early patients), while the diagnosis of MSA was made according to the 2008 criteria of the Second consensus statement(Wenning et al. 2022).

Fourteen PD patients (14/23) and all MSA patients gave consent to execution of skin biopsies to provide biological characterization.

Genetic analysis was carried out on a standardized set of genes (including *SNCA, LRRK2, GBA1, PRKN, PINK1, DJ1, VPS13 C, SYNJ1, VPS35, ATP13 A2, DNAJC6, FBXO7, PLA2G6, CHCHD2*; genes associated with parkinsonism with a possible role in PD susceptibility or phenocopies such as *GRN, MAPT, DCTN1, NPC1, NPC2, POLG, GCH1, ATP1 A3*; research PD genes such as *CSMD1, LRP10, TWNK, EIF4G1, DNACJ13, UCHL1*) (Bonato et al. 2024; Carrer et al. 2024).

All PD and MSA patients underwent OCT, with one MSA patient being eventually excluded for the poor quality of the images. OCT data were compared with normative parameters established in a healthy population of European background by Motamedi et al. (Motamedi et al. 2019). Exclusion criteria for performing OCT were severe myopia, hyperopia or astigmatism, previous intraocular surgery, or coexisting ophthalmological conditions (i.e., age-related macular or retinal degeneration, glaucoma, cataract).

Ten healthy subjects (4 males, mean age 51.3 ± 8.5 yrs) with no evidence of neurological disorders were considered as healthy controls for the hyperreflective intraretinal foci (HRF) counting.

- a. Clinical evaluation
  All patients underwent standard neurological examination using specific validated clinical rating scales, namely the Italian version of the MDS-Unified Parkinson’s Disease Rating Scale (MDS-UPDRS) for PD patients^21^, and the Unified Multiple System Atrophy Rating Scale (UMSARS) for MSA patients^22^. Moreover, the presence of possible dysautonomia was assessed employing a specific questionnaire, the Composite Autonomic Symptom Score (COMPASS 31) (Sletten et al. 2012). COMPASS 31 total score and the sub-scores corresponding to the six different domains [OH, vasomotor, secretomotor, gastrointestinal (GI), genitourinary (GU) and pupillomotor] were calculated. Global Cognitive function was assessed using the Montreal Cognitive Assessment (MoCA) (Fiorenzato et al. 2019).
- b. Skin biopsies
  Two skin punch-biopsies (ø = 3 mm; depth 3 ± 2 mm) were performed in 14/23 PD patients and in all 12 MSA patients in the cervical C7 paravertebral area after injecting lidocaine to ensure local anaesthesia. Sampled biopsies were collected in sterile Phosphate Buffered Solution (PBS) and subsequently underwent histopathological evaluation via immunohistochemistry (IHC).
  Biopsies were fixed for 2448 h at 4 °C in Zamboni solution, paraffin-embedded and sectioned as 5µm thick sections at the rotary microtome (Leica RM2155) according to a previously published protocol (Campagnolo et al. 2024). Haematoxylin and Eosin staining was employed for routine histopathological evaluation. Immunoperoxidase staining was performed on a Dako EnVision Autostainer (Dako Denmark A/S, Glostrup, Denmark) according to manufacturer recommendations. Antibodies for phosphorylated alpha-synuclein Ser129 (Monoclonal Rabbit, dilution 1:1000), aggregated αSyn (Monoclonal Mouse, Clone 5G4, dilution 1:20.000, Millipore) and PGP9.5 (Polyclonal Rabbit, dilution 1:300, Abcam; Monoclonal Mouse, dilution 1:1000, Thermo-Fisher) were employed for single- and double-label immunoperoxidase staining, as well as immunofluorescent staining. Antigen retrieval was performed on a PT-Link Dako Antigen retrieval station using Citrate-buffer at pH 6 solution at 96° for 15 min, followed by 1 min 95% formic acid for the αSyn- 5G4 antibody. Slides were evaluated by an experienced pathologist and accordingly scored. Immunoperoxidase staining was performed on a Dako EnVision Autostainer (Dako Denmark A/S, Glostrup, Denmark) according to manufacturer recommendations and in accordance to previously established protocols (Emmi et al. 2023). Immunoperoxidase staining was repeated at least three times to ensure reaction consistency. Fluorescent immunohistochemistry was performed manually. Antigen retrieval was performed on de-paraffinized tissue as in immunoperoxidase staining methods. Following autofluorescence was quenched with a 50 mM NH4 Cl solution for 10 min. Sections were treated with permeabilization and blocking solution (15% vol/vol Goat Serum, 2% wt/vol BSA, 0.25% wt/vol gelatin, 0.2% wt/vol glycine in PBS) containing 0.5% Triton X100 for 90 min before primary antibodies incubation. Primary antibodies were diluted in blocking solution and incubated at 4 °C overnight. Alexa-Fluor plus 488 Goat anti-Mouse secondary antibody (A32723, Thermo Fisher Scientific) and Alexa-Fluor plus 568 anti-Rabbit secondary antibody (A- 11011, Thermo Fisher Scientific) were diluted 1:200 in blocking solution as above and incubated for 60 minutes at room temperature. Hoechst 33258 were used for nuclear staining (Invitrogen, dilution: 1:10000 in PBS) for 10 minutes. Slides were mounted and coverslipped with Mowiol solution (Novabiochem). Confocal immunofluorescence z-stack images were acquired on a Leica SP5 Laser Scanning Confocal Microscope using a HC PL FLUOTAR 20x/0.50 Dry or HCX PL APO lambda blue 40X/1.40 Oil objectives. Images were acquired at a 16-bit intensity resolution over 2048 × 2048 pixels. Z-stacks images were converted into digital maximum intensity z-projections, processed, and analyzed using ImageJ software. IHC scoring: each biopsy was given a score ranging from 0 (no evidence of synucleinopathy) to 1. Scoring was based on four factors: phosphorylated alpha-synuclein deposition in vascular innervation (1), gland innervation (2), free nerve endings (3) and large dermal/hypodermal nerve fibers (4). Each factor was assigned a score of 0 (negative) and 1 (positive); the patient was considered positive for synucleinopathy if average score among the four factors was > 0.25 (i.e., at least one factor had a score of 1).
- c. OCT
  All subjects underwent OCT imaging using SPECTRALIS® HRA+OCT (Heidelberg Engineering, Heidelberg, Germany), that combines SD-OCT technology with laser scanning confocal ophthalmoscopy (cSLO) with infrared wave (IR, 820 nm) according to a previously reported protocol. TruTrack^™^ Active Eye Tracking, a patented technology that uses a second laser beam to actively track the eye during OCT scanning to avoid motion artifact, was used. The result is a punctual correlation between fundus oculi and OCT scans, associated with improvement of image definition and sharpness. Both eyes (OD and OS) were examined by experts, in a dim light and without the use of mydriatic agents.

The protocol included:

- A 20 × 20° volumetric macular scan automatically centred on the fovea and obtained with 25 vertical B-scans (distance between B-scans 240 µm), ART 49. The software allows the evaluation of the macular volume total (VM), calculated as the volume subtended by a surface area defined by a circle having the fovea as its centre and a radius of 2 mm. The internationally recognized 9-segment ETDRS (Early Treatment Diabetic Retinopathy Screening) map subfield measurements were performed using the inbuilt Spectralis mapping software. Measurements are automatically averaged across each of the following subfields and sectors: the central fovea subfield within the inner 1-mm-diameter circle; the inner circle subfield between the inner and middle 3-mm-diameter circles; and the outer circle subfield between the middle and outer 6-mm-diameter circles. Both the inner and the outer circles were sectioned into superior, nasal, inferior, and temporal quadrants. For each macular scan, the Spectralis software automatically segmented the different retinal layers (RNFL, GCL, IPL, INL, OPL, ONL, RPE). The following parameters of the macular scans were considered: total volume of retinal layers RNFL, GCL, IPL, INL, OPL, ONL, RPE, IRL (comprising the RNFL, GCL, IPL and INL layers) and ORL (outer retinal layers, comprising the OPL, ONL and RPE layers), total retinal volume, and total thickness of the retina and retinal layers. The inner ring sectors (i.e., S1, I1, N1, T1) were grouped into a single data (IR) representing the average among the inner ring thicknesses, and the same was done with the outer ring sectors; the thickness values in S2, I2, N2, T2 were averaged to obtain the OR data. This was done to overcome the problem of specularity between nasal and temporal sectors between the two eyes. Each SD-OCT scan was then re-evaluated by an experienced neurologist, in order to apply, if necessary, a manual correction of the segmentation, thus ensuring the accuracy of the stratification. Low quality scans (< 15 dB) according to OSCAR-IB criteria (Tewarie et al. 2012) were not included in the analysis. Z score of LR RNFL, GC-IPL, INL and total retina thickness were obtained based on normative mean and SD value of the normal population of European background (218 healthy volunteers (144 females, age 36.5 ± 12.3 years, range 18–69 years) (Motamedi et al. 2019). Atrophy was defined if Z score were below − 1 SD the normal mean, while hypertrophy was defined for measure above 1 SD the normal mean.
- Hyperreflective intraretinal foci (HRF) counting was performed, according to the protocol described in previous publications (Pengo et al. 2022; Pilotto et al. 2019), employing the analysis of the central linear scan of the macular map, crossing the fovea. HRF were identified as isolated punctiform elements of small dimensions (≤ 30μm) with intermediate reflectivity (similar to the RNFL reflectivity) and without a shadow cone. HRF were counted in the central 3 mm, included between two perpendicular lines to Bruch’s Membrane traced at 1500 μm both temporally and nasally from the centre of the fovea. The count was performed by consensus by two observers blinded (MP and EB) to the diagnosis, and divided into GCL, IPL and INL.

### Statistical analysis

Clinical and demographical continuous and dichotomous variables differences between PD and MSA subgroups were compared with Mann Whitney U test and Fisher test respectively. A Bonferroni adjusted threshold p < 0.002 was considered. To evaluate the clinical usefulness of OCT assessment within five years at patient level, the percentage of alteration (atrophy/hypertrophy) was calculated in the MSA and PD sample based on Z score of LR RNFL, GC-IPL, INL and total retina thickness. A percentage of 90% was considered clinically meaningful and measures that reached this percentage were considered for further analyses. LR z score OCT central, inner, and outer temporal, nasal, inferior, and superior measures profile were compared between PD and MSA subgroups with Mann Whitney U test. A Bonferroni adjusted threshold p < 0.0055 was considered. A dot&line diagram was used to compare the single patient OCT profile in PD and MSA subgroup. To rule out the possible effect of age confound Ancova analysis including age at visit in the model was run comparing mRNFL, mGCIPL and mINL between HC, PD and MSA. Bootstrapping with 500 repetition was adopted to improve population representativeness and mitigate heteroscedasticity. Partial Spearman correlation conditioned on variable age at visit was run separately in MSA and PD to assess motor, cognitive and autonomic correlation with significant markers. The HRF data were evaluated by analysis of covariance (ANCOVA) performed by the Kruskal–Wallis nonparametric test, followed by post-hoc comparisons test. All analyses were done with SPSS version 25.0.

## Results

Overall, 35 patients were enrolled in the study: twenty-three PD patients (16 males, median age 67 yrs, range 49.4–76.8 yrs) and a median disease duration of 2.3 yrs (range 0.3–5.1 yrs) and twelve MSA patients (4 males, median age 66.5 yrs, range 50–81) with a median disease duration (considered at the time of the evaluation) of 4.8 yrs (range 3.3–5.8 yrs).

All patients presented negative genetic testing, except for one patient disclosing a pathogenic granulin precursor (GRN) mutation and clinical features consistent with PD (asymmetric bradykinesia and rigidity with preserved cognitive function after 4-year of follow-up).

No significant differences in median age were found when comparing the PD and MSA subgroups. Disease duration was longer in MSA patients (p = 0.00022).

Demographic data are reported in Table 1.

**Table 1.**
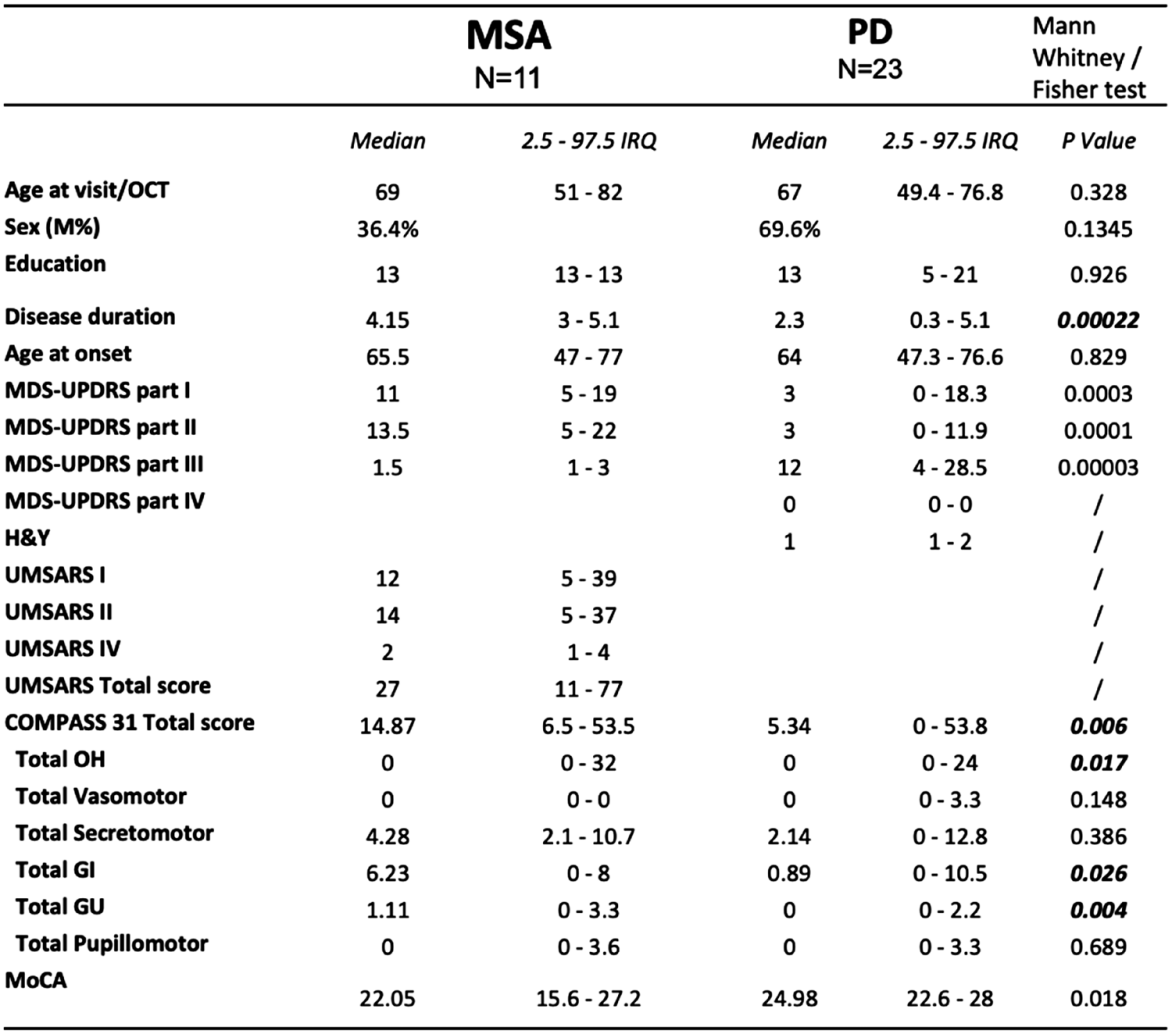
Demographic and clinical data of the PD and MSA cohorts

*MDS-UPDRS* MDS-unified Parkinson’s disease rating scale, *UMSARS* unified multiple system atrophy rating scale, *COMPASS 31* the composite autonomic symptom score 31 questionnaire, *Moca* montreal cognitive assessment

A Bonferroni adjusted threshold p < 0.002 was considered

### Clinical evaluation

In PD patients the median scores were 3 (range 0–18.3) for the MDS-UPDRS I, 3 (range 0–11.9) for the MDS-UPDRS II, 12 (range 4–28.5) for the MDS-UPDRS III.

In MSA UMSARS median total score was 27 (range 11–77). The median scores in the different sections of the scale were as follows: UMSARS I 12 (range 5–39), UMSARS II 14 (range 5–37) and UMSARS IV 2 (range 1–4).

The presence of dysautonomia was evaluated in PD and MSA patients using the COMPASS 31 score. In PD patients, the COMPASS 31 median scores in the different domains were as follows: OH 0 (range 0–24), vasomotor 0 (range 0–3.3.), secretomotor 2.14 (range 0–12.8), GI 0.89 (range 0–10.5), GU 0 (range 0–2.2), pupillomotor 0 (range 0–3.3.). The median COMPASS 31 total score in PD patients was 5.34 (range 0–53.8). In MSA patients, the median scores in the different domains were as follows: OH 0 (range 0–32), vasomotor 0, secretomotor 4.28 (range 2.1–10.7), GI 6.23 (range 0–8), GU 1.1 (range 0–3.3.), pupillomotor 0 (range 0–3.6). The median COMPASS 31 total score in MSA patients was 14.87 (range 6.5–53.5).

A statistically significant difference was observed when comparing in PD vs MSA COMPASS 31 total scores (5.34 with range 0–53.8 vs 14.87 with range 6.5–53.5 respectively, p = 0.006), with MSA patients disclosing higher scores in the OH, GI and GU domains (p = 0.017, p = 0.026, p = 0.004 respectively).

Clinical data are reported in Table 1.

### OCT

As detailed in the Methods section, OCT data were compared with normative parameters established in a healthy population of European background by Motamedi et al. (2019).

No significant differences were reported in the mean global retinal thickness between PD, MSA and healthy controls (313.2 ± 35.3 in PD, 312.3 ± 10.6 in MSA, 321.7 ± 11.5 in HC respectively). A significant reduction in the RNFL mean global thickness was observed in PD and MSA patients when compared with controls (28.7 ± 4.6 in PD, 27.5 ± 2.4 in MSA, 33.5 ± 2.7 in HC, respectively, p < 0.001) with no difference disclosed between PD and MSA.

Regarding GCL + IPL, the mean global thickness resulted significantly reduced in MSA when compared with both PD and HC (31.2 ± 8.8 vs 44.6 ± 16.1 and 74.9 ± 4.8 respectively, p < 0.001). Moreover, the GCL + IPL mean thickness was also significantly reduced in PD when compared with HC (44.6 ± 16.1 vs 74.9 ± 4.8, p < 0.001).

**Fig. 1:**
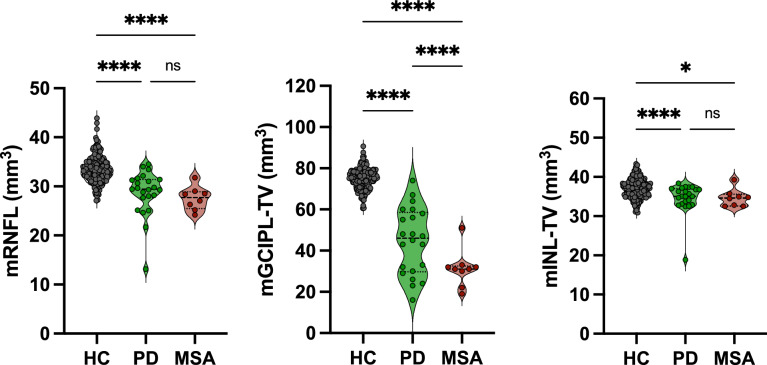
Differences in mean global thickness across different retinal layers in patients (PD, MSA) and in healthy controls

INL mean global thickness was significantly reduced only when considering PD in comparison with HC (34.6 ± 3.9 vs 37.1 ± 2.4, p = 0.02) (Fig. 1) (Table 2) (eFig 1, Supplementary Materials).

**Table 2.**
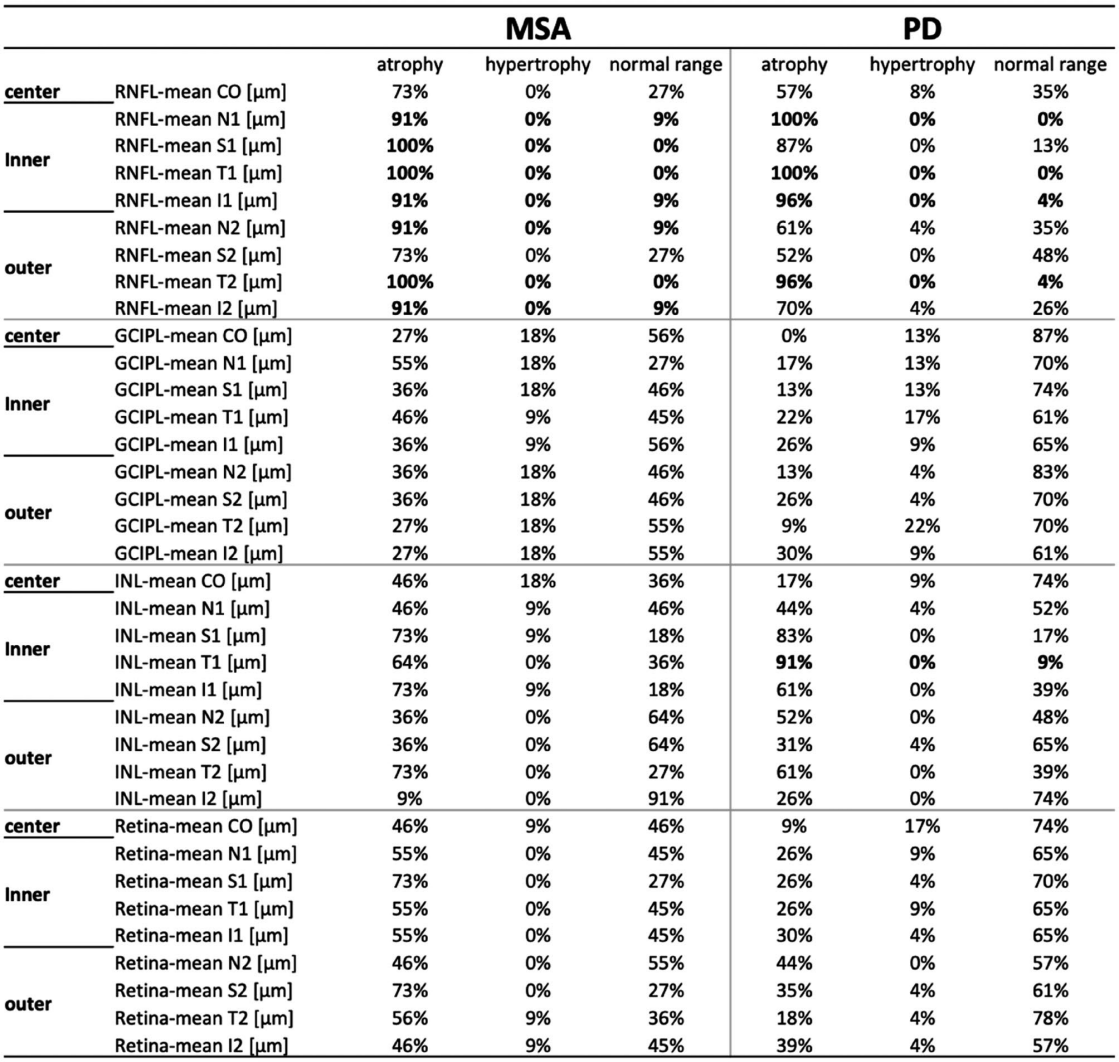
Percentage of disease-specific OCT abnormalities in PD and MSA patients

Z scores of RNFL, GCIPL, INL and total retina thickness were obtained based on normative mean and standard deviation (SD) values in the normal Caucasian population. Atrophy was defined if Z scores were below − 1 SD the normal mean, while hypertrophy was defined for measures above 1 SD the normal mean. A percentage of 90% was considered clinically meaningful

Regarding RNFL, we found sectorial atrophy involving the inner nasal/temporal/inferior sectors and the outer temporal section in PD patients whereas MSA patients disclosed a preferential involvement of the inner superior and outer nasal/inferior RNFL sectors. Overall, a significant percentage of atrophy has been measured in each RNFL sector in both MSA and PD. GCL + IPL did not disclose a specific sectorial atrophy pattern in PD and MSA patients, whereas the INL resulted significantly atrophic in the inner superior and temporal sectors in PD patients.

No OCT measure showed a statistically significant correlation with disease severity and clinical (including autonomic, cognitive and motor) scores in either the PD or MSA cohort (eFig 2, Supplementary Materials).

Regarding the HRF counting, 10 healthy subjects (4 males, mean age 51.3 ± 8.5 yrs) with no evidence of neurological disorders were considered. HRF counting was performed in the INL, in the GCIP (GCL + IPL) and in the IRL (INL + GCL + IPL). The overall number of HRF was higher in the patients’ subgroup when compared to healthy controls, with PD patients presenting a higher number of HRF than MSA patients (p < 0.001) (Table 1, Supplementary Materials).

### Skin biopsies

IHC mean score was 0.32 ± 0.3 in PD and 0.27 ± 0.27 in MSA.

The anatomical localization of pathological α-syn was also investigated, with the majority of PD patients showing predominant deposition in the vascular nerves, as opposed to MSA patients, disclosing an even distribution among vascular, glandular and hypodermal/dermal nerves.

Data regarding skin biopsies are reported in the Supplementary Materials (Tables 2, 3).

## Discussion

In this study, we investigated OCT parameters in early PD and MSA. Consistent with previous literature, the majority of our patients exhibited abnormalities in the Retinal Nerve Fiber Layer (RNFL), displaying sectorial atrophy patterns that varied between subgroups. Specifically, PD patients showed atrophy in inner nasal/temporal/inferior and outer temporal sections, whereas MSA patients displayed atrophy in inner superior and outer nasal/inferior sectors. Our findings in PD revealed a significant reduction in the RNFL thickness compared to age and gender-matched controls. This reduction predominantly affected the inferior, superior, and temporal RNFL regions, while sparing the nasal sector (Chrysou et al. 2019; Huang et al. 2021). Previous studies have presented conflicting results, potentially due to small sample sizes and technical differences. Many studies reported no changes in PD vs. healthy controls, and this included the INL parameters^31−35^. The INL is particularly relevant in PD, since it constitutes the primary location where dopaminergic cells are located, with axons projecting to the GCL + IPL. All patients included in our PD cohort had short disease duration and presented significant INL atrophy that was not observed in the MSA cohort. This difference, however, was not statistically significant, suggesting that although being potentially useful as a biomarker in the early stages of PD, the INL atrophy pattern should be further investigated and confirmed in larger cohorts, in order to corroborate its role in the differential diagnosis of α-synucleinopathies. In line with findings in prior studies, significant global atrophy was also observed in the GCL + IPL, with no evidence of sectorial abnormalities, possibly due to the inclusion of a homogeneous cohort of PD with short disease duration (Chan et al. 2019; Wagner et al. 2023) 

The application of OCT in the diagnosis and follow-up of α-synucleinopathies may be particularly important given the strong evidence suggesting that retinal pathology may mirror brain pathology in PD (Ahn et al. 2016). Diffuse deposition of pathological α-syn in the inner retinal layers has been observed (Ortuño-Lizarán et al. 2018), showing a significant correlation with clinical data, particularly disease severity (Ortuño-Lizarán et al. 2018, 2020). In line with that, visual dysfunction (particularly impaired contrast sensitivity and acuity) is a common complaint in PD patients, that contributes to worsen visuospatial deficits and may contribute to visual hallucinations, especially in advanced PD patients (Lee et al. 2022; Ortuño-Lizarán et al. 2020; Murueta-Goyena et al. 2019). In our cohort, no OCT measure showed a statistically significant correlation with disease severity and clinical (i.e. autonomic, cognitive, motor) scores, possibly due to the small sample size and to the fact that all patients were classified as “early PDs” and none reported visual symptoms. Possible correlations of OCT findings with clinical features are still under investigation, although strong evidence has been observed between cognitive impairment and RNFL and GCL + IPL atrophy (Zhang et al. 2021; Murueta-Goyena et al. 2021).

The presence of retinal abnormalities, with predominant atrophy involving the inferior, superior and nasal RNFL sectors in MSA has been reported by other studies, although not consistently (Mendoza-Santiesteban et al. 2017; Ma et al. 2023; Albrecht et al. 2012; Fischer et al. 2013).

OCT also offers insight into HRF, which have been extensively studied in various neuro-ophthalmological conditions. HRF are characterized by small (< 30 μm), hyperreflective, round lesions observable in every retinal layer. While their potential as biomarkers has been primarily explored in MS, where an association between HRF count and cortical pathology has been noted, research in synucleinopathies is scarce (Pengo et al. 2022; Pilotto et al. 2019). HRF consist of clusters of activated and proliferating microglia, sharing morphological and functional similarities with CNS microglia (Vujosevic et al. 2017; Choi et al. 2021). These cells play a pivotal role in the pathogenesis of inflammatory and neurodegenerative diseases, contributing to an inflammatory milieu conducive to α-synuclein misfolding and deposition in PD (Garcia et al. 2022; Vieira et al. 2015). In our cohort, HRF counts were elevated across all retinal layers in both PD and MSA compared to healthy controls, with PD patients exhibiting the highest values. These findings may lead to the assumption that a greater retinal damage due to α-synuclein pathology and determining microglial activation is occurring. However, the inconsistencies in the HRF count across different retinal layers and the absence of solid data in synucleinopathies prompts further evaluations in order to define the possible role of this parameter in neurodegenerative disorders.

We recognize that our study has limitations. Our sample size was relatively small, although we meticulously selected patients based on validated clinical criteria and providing genetic and biological support to the diagnosis. We used stringent criteria to ensure high-quality OCT imaging by excluding patients with ophthalmological conditions such as cataracts. Therefore, our findings cannot be immediately translated to the whole PD and MSA population. As reported in the Methods section, we compared our OCT data with normative parameters established in a healthy population of European background (Motamedi et al. 2019), whereas a control group of individuals with other neurodegenerative diseases was not evaluated. This would widen the knowledge regarding the application of OCT as an additional diagnostic tool in these conditions.

We believe our results encourage further research in OCT imaging in α-synucleinopathies, focusing on larger cohorts and newly diagnosed patients where the biological characterization becomes especially important in light of the availability of potentially disease-modifying therapies. OCT features (particularly INL abnormalities and HRF counts) might potentially consolidate as valuable biomarkers, both in the diagnostic work-up and in the differential diagnosis of α-synucleinopathies.

## Supplementary Information

Below is the link to the electronic supplementary material.

Supplementary file1 (DOCX 412 kb)

## Data Availability

All data collected or analyzed during this study are included (see manuscript and Supplementary Materials).

## References

[CR1] Ahn J, Lee JY, Kim TW (2016) Retinal thinning correlates with clinical severity in multiple system atrophy. J Neurol 263:2039–2047. 10.1007/s00415-016-8230-0 27416856 10.1007/s00415-016-8230-0

[CR2] Albrecht P, Müller AK, Südmeyer M, Ferrea S, Ringelstein M, Cohn E, Aktas O, Dietlein T, Lappas A, Foerster A, Hartung HP, Schnitzler A, Methner A (2012) Optical coherence tomography in parkinsonian syndromes. PLoS ONE 7(4):e34891. 10.1371/journal.pone.0034891 22514688 10.1371/journal.pone.0034891 PMC3325949

[CR3] Antonini A, Abbruzzese G, Ferini-Strambi L, Tilley B, Huang J, Stebbins GT, Goetz CG, Barone P, MDS-UPDRS Italian Validation Study Group, di Bandettini Poggio M, Fabbrini G, Di Stasio F, Tinazzi M, Bovi T, Ramat S, Meoni S, Pezzoli G, Canesi M, Martinelli P, Maria Scaglione CL, Rossi A, Tambasco N, Santangelo G, Picillo M, Morgante L, Morgante F, Quatrale R, Sensi M, Pilleri M, Biundo R, Nordera G, Caria A, Pacchetti C, Zangaglia R, Lopiano L, Zibetti M, Zappia M, Nicoletti A, Quattrone A, Salsone M, Cossu G, Murgia D, Albanese A, Del Sorbo F (2013) Validation of the Italian version of the movement disorder society-unified Parkinson’s disease rating scale. Neurol Sci 34(5):683–687. 10.1007/s10072-012-1112-z 22678179 10.1007/s10072-012-1112-z

[CR4] Bittersohl D, Stemplewitz B, Keserü M, Buhmann C, Richard G, Hassenstein A (2015) Detection of retinal changes in idiopathic Parkinson’s disease using high-resolution optical coherence tomography and heidelberg retina tomography. Acta Ophthalmol 93(7):e578–e584. 10.1111/aos.12757 26267660 10.1111/aos.12757

[CR5] Bonato G, Antonini A, Pistonesi F, Campagnolo M, Guerra A, Biundo R, Pilleri M, Bertolin C, Salviati L, Carecchio M (2024) Genetic mutations in Parkinson’s disease: screening of a selected population from North-Eastern Italy. Neurol Sci. 10.1007/s10072-024-07690-7 39034353 10.1007/s10072-024-07690-7 PMC11698772

[CR6] Campagnolo M, Weis L, Sandre M, Tushevski A, Russo FP, Savarino E, Carecchio M, Stocco E, Macchi V, De Caro R, Parchi P, Bubacco L, Porzionato A, Antonini A, Emmi A (2024) Immune landscape of the enteric nervous system differentiates Parkinson’s disease patients from controls: The PADUA-CESNE cohort. Neurobiol Dis 200:106609. 10.1016/j.nbd.2024.106609 39048026 10.1016/j.nbd.2024.106609

[CR7] Carrer T, Bonato G, Sandre M, Emmi A, Campagnolo M, Musso G, Carecchio M, Parchi P, Antonini A (2024) Rapidly progressive multiple system atrophy in a patient carrying LRRK2 G2019S mutation. Neurol Sci 45(1):309–313. 10.1007/s10072-023-07056-5 37752324 10.1007/s10072-023-07056-5

[CR8] Chan VTT, Sun Z, Tang S, Chen LJ, Wong A, Tham CC, Wong TY, Chen C, Ikram MK, Whitson HE, Lad EM, Mok VCT, Cheung CY (2019) Spectral-domain OCT measurements in Alzheimer’s disease: a systematic review and meta-analysis. Ophthalmology 126(4):497–510. 10.1016/j.ophtha.2018.08.009 30114417 10.1016/j.ophtha.2018.08.009 PMC6424641

[CR9] Choi S, Guo L, Cordeiro MF (2021) Retinal and brain microglia in multiple sclerosis and neurodegeneration. Cells 10:1507. 10.3390/cells10061507 34203793 10.3390/cells10061507 PMC8232741

[CR10] Chopra A, Lang AE, Höglinger G, Outeiro TF (2024) Towards a biological diagnosis of PD. Parkinsonism Relat Disord 122:106078. 10.1016/j.parkreldis.2024.106078 38472075 10.1016/j.parkreldis.2024.106078

[CR11] Chrysou A, Jansonius NM, van Laar T (2019) Retinal layers in Parkinson’s disease: a meta-analysis of spectral-domain optical coherence tomography studies. Parkinsonism Relat Disord 64:40–49. 10.1016/j.parkreldis.2019.04.023 31054866 10.1016/j.parkreldis.2019.04.023

[CR12] Donadio V, Incensi A, Rizzo G, De Micco R, Tessitore A, Devigili G, Del Sorbo F, Bonvegna S, Infante R, Magnani M, Zenesini C, Vignatelli L, Cilia R, Eleopra R, Tedeschi G, Liguori R (2020) Skin biopsy may help to distinguish multiple system atrophy-Parkinsonism from Parkinson’s disease with orthostatic hypotension. Mov Disord 35(9):1649–1657. 10.1002/mds.28126 32557839 10.1002/mds.28126

[CR13] Donadio V, Wang Z, Incensi A, Rizzo G, Fileccia E, Vacchiano V, Capellari S, Magnani M, Scaglione C, Stanzani Maserati M, Avoni P, Liguori R, Zou W (2021) In vivo diagnosis of synucleinopathies: a comparative study of skin biopsy and RT-QuIC. Neurology 96(20):e2513–e2524. 10.1212/WNL.0000000000011935 33837116 10.1212/WNL.0000000000011935 PMC8205473

[CR14] Emmi A, Sandre M, Russo FP, Tombesi G, Garrì F, Campagnolo M, Carecchio M, Biundo R, Spolverato G, Macchi V, Savarino E, Farinati F, Parchi P, Porzionato A, Bubacco L, De Caro R, Kovacs GG, Antonini A (2023) Duodenal alpha-synuclein pathology and enteric gliosis in advanced Parkinson’s Disease. Mov Disord 38(5):885–894. 10.1002/mds.29358 36847308 10.1002/mds.29358

[CR15] Fiorenzato E, Antonini A, Camparini V, Weis L, Semenza C, Biundo R (2019) Characteristics and progression of cognitive deficits in progressive supranuclear palsy vs. multiple system atrophy and Parkinson’s disease. J Neural Transm (Vienna) 126(11):1437–1445. 10.1007/s00702-019-02065-1 31432258 10.1007/s00702-019-02065-1

[CR16] Fischer MD, Synofzik M, Kernstock C, Dietzsch J, Heidlauf R, Schicks J, Srulijes K, Wiethoff S, Menn O, Berg D, Schöls L, Schiefer U (2013) Decreased retinal sensitivity and loss of retinal nerve fibers in multiple system atrophy. Graefes Arch Clin Exp Ophthalmol 251(1):235–241. 10.1007/s00417-012-2118-1 22878471 10.1007/s00417-012-2118-1

[CR17] Garcia P, Jürgens-Wemheuer W, Uriarte Huarte O, Michelucci A, Masuch A, Brioschi S, Weihofen A, Koncina E, Coowar D, Heurtaux T, Glaab E, Balling R, Sousa C, Kaoma T, Nicot N, Pfander T, Schulz-Schaeffer W, Allouche A, Fischer N, Biber K, Kleine-Borgmann F, Mittelbronn M, Ostaszewski M, Schmit KJ, Buttini M (2022) Neurodegeneration and neuroinflammation are linked, but independent of alpha-synuclein inclusions, in a seeding/spreading mouse model of Parkinson’s disease. Glia 70:935–960. 10.1002/glia.24149 35092321 10.1002/glia.24149 PMC9305192

[CR18] Gibbons C, Wang N, Rajan S, Kern D, Palma JA, Kaufmann H, Freeman R (2023) Cutaneous α-synuclein signatures in patients with multiple system atrophy and Parkinson disease. Neurology 100(15):e1529–e1539. 10.1212/WNL.0000000000206772 36657992 10.1212/WNL.0000000000206772 PMC10103107

[CR19] Gibbons CH, Levine T, Adler C, Bellaire B, Wang N, Stohl J, Agarwal P, Aldridge GM, Barboi A, Evidente VGH, Galasko D, Geschwind MD, Gonzalez-Duarte A, Gil R, Gudesblatt M, Isaacson SH, Kaufmann H, Khemani P, Kumar R, Lamotte G, Liu AJ, McFarland NR, Miglis M, Reynolds A, Sahagian GA, Saint-Hillaire MH, Schwartzbard JB, Singer W, Soileau MJ, Vernino S, Yerstein O, Freeman R (2024) Skin biopsy detection of phosphorylated α-synuclein in patients with synucleinopathies. JAMA 331(15):1298–1306. 10.1001/jama.2024.0792 38506839 10.1001/jama.2024.0792 PMC10955354

[CR20] Huang L, Zhang D, Ji J, Wang Y, Zhang R (2021) Central retina changes in Parkinson’s disease: a systematic review and meta-analysis. J Neurol 268(12):4646–4654. 10.1007/s00415-020-10304-9 33174132 10.1007/s00415-020-10304-9

[CR21] Lee JY, Martin-Bastida A, Murueta-Goyena A, Gabilondo I, Cuenca N, Piccini P, Jeon B (2022) Multimodal brain and retinal imaging of dopaminergic degeneration in Parkinson disease. Nat Rev Neurol 18(4):203–220. 10.1038/s41582-022-00618-9 35177849 10.1038/s41582-022-00618-9

[CR22] London A, Benhar I, Schwartz M (2013) The retina as a window to the brain-from eye research to CNS disorders. Nat Rev Neurol 9(1):44–53. 10.1038/nrneurol.2012.227 23165340 10.1038/nrneurol.2012.227

[CR23] Ma X, Li S, Zheng B, Hu L, Liu H, Wang Z, Wang Z, Chen H, Su W (2023) Retinal structure abnormalities in Parkinson’s disease and atypical Parkinsonism. Biomolecules 13(2):218. 10.3390/biom13020218 36830588 10.3390/biom13020218 PMC9952897

[CR24] Magalhães P, Lashuel HA (2022) Opportunities and challenges of alpha-synuclein as a potential biomarker for Parkinson’s disease and other synucleinopathies. NPJ Parkinsons Dis 8(1):93. 10.1038/s41531-022-00357-0 35869066 10.1038/s41531-022-00357-0 PMC9307631

[CR25] Mailankody P, Battu R, Khanna A, Lenka A, Yadav R, Pal PK (2015) Optical coherence tomography as a tool to evaluate retinal changes in Parkinson’s disease. Parkinsonism Relat Disord 21(10):1164–1169. 10.1016/j.parkreldis.2015.08.002 26297381 10.1016/j.parkreldis.2015.08.002

[CR26] McCann H, Stevens CH, Cartwright H, Halliday GM (2014) α-synucleinopathy phenotypes. Parkinsonism Relat Disord 20(Suppl 1):S62–S67. 10.1016/S1353-8020(13)70017-8 24262191 10.1016/S1353-8020(13)70017-8

[CR27] Mendoza-Santiesteban CE, Gabilondo I, Palma JA, Norcliffe-Kaufmann L, Kaufmann H (2017) The retina in multiple system atrophy: systematic review and meta-analysis. Front Neurol 8:206. 10.3389/fneur.2017.00206 28596752 10.3389/fneur.2017.00206 PMC5443142

[CR28] Mirmosayyeb O, Zivadinov R, Weinstock-Guttman B, Benedict RHB, Jakimovski D (2023) Optical coherence tomography (OCT) measurements and cognitive performance in multiple sclerosis: a systematic review and meta-analysis. J Neurol 270(3):1266–1285. 10.1007/s00415-022-11449-5 36396812 10.1007/s00415-022-11449-5

[CR29] Motamedi S, Gawlik K, Ayadi N, Zimmermann HG, Asseyer S, Bereuter C, Mikolajczak J, Paul F, Kadas EM, Brandt AU (2019) Normative data and minimally detectable change for inner retinal layer thicknesses using a semi-automated OCT image segmentation pipeline. Front Neurol 10:1117. 10.3389/fneur.2019.01117 31824393 10.3389/fneur.2019.01117 PMC6886563

[CR30] Murueta-Goyena A, Del Pino R, Reyero P, Galdós M, Arana B, Lucas-Jiménez O, Acera M, Tijero B, Ibarretxe-Bilbao N, Ojeda N, Peña J, Cortés J, Gómez-Esteban JC, Gabilondo I (2019) Parafoveal thinning of inner retina is associated with visual dysfunction in Lewy body diseases. Mov Disord 34(9):1315–1324. 10.1002/mds.27728 31136022 10.1002/mds.27728 PMC6790692

[CR31] Murueta-Goyena A, Del Pino R, Galdós M, Arana B, Acera M, Carmona-Abellán M, Fernández-Valle T, Tijero B, Lucas-Jiménez O, Ojeda N, Ibarretxe-Bilbao N, Peña J, Cortes J, Ayala U, Barrenechea M, Gómez-Esteban JC, Gabilondo I (2021) Retinal thickness predicts the risk of cognitive decline in Parkinson disease. Ann Neurol 1:165–176. 10.1002/ana.25944 10.1002/ana.25944 PMC7756646 33098308

[CR32] Ortuño-Lizarán I, Beach TG, Serrano GE, Walker DG, Adler CH, Cuenca N (2018) Phosphorylated α-synuclein in the retina is a biomarker of Parkinson’s disease pathology severity. Mov Disord 33(8):1315–1324. 10.1002/mds.27392 29737566 10.1002/mds.27392 PMC6146055

[CR33] Ortuño-Lizarán I, Sánchez-Sáez X, Lax P, Serrano GE, Beach TG, Adler CH, Cuenca N (2020) Dopaminergic retinal cell loss and visual dysfunction in Parkinson disease. Ann Neurol 88(5):893–906. 10.1002/ana.25897 32881029 10.1002/ana.25897 PMC10005860

[CR34] Outeiro TF, Alcalay RN, Antonini A, Attems J, Bonifati V, Cardoso F, Chesselet MF, Hardy J, Madeo G, McKeith I, Mollenhauer B, Moore DJ, Rascol O, Schlossmacher MG, Soreq H, Stefanis L, Ferreira JJ (2023) Defining the riddle in order to solve it: there is more than one “Parkinson’s disease.” Mov Disord 38(7):1127–1142. 10.1002/mds.29419 37156737 10.1002/mds.29419

[CR35] Peng H, Chen S, Wu S, Shi X, Ma J, Yang H, Li X (2023) Alpha-synuclein in skin as a high-quality biomarker for Parkinson’s disease. J Neurol Sci 451:120730. 10.1016/j.jns.2023.120730 37454572 10.1016/j.jns.2023.120730

[CR36] Pengo M, Miante S, Franciotta S, Ponzano M, Torresin T, Bovis F, Rinaldi F, Perini P, Saiani M, Margoni M, Bertoldo A, Sormani MP, Pilotto E, Midena E, Gallo P, Puthenparampil M (2022) Retinal hyperreflecting foci associate with cortical pathology in multiple sclerosis. Neurol Neuroimmunol Neuroinflamm 9(4):e1180. 10.1212/NXI.0000000000001180 35606113 10.1212/NXI.0000000000001180 PMC9128002

[CR37] Pilotto E, Leonardi F, Stefanon G, Longhin E, Torresin T, Deganello D, Cavarzeran F, Miglionico G, Parrozzani R, Midena E (2019) Early retinal and choroidal OCT and OCT angiography signs of inflammation after uncomplicated cataract surgery. Br J Ophthalmol 103(7):1001–1007. 10.1136/bjophthalmol-2018-312461 30127073 10.1136/bjophthalmol-2018-312461

[CR38] Postuma RB, Berg D, Stern M, Poewe W, Olanow CW, Oertel W, Obeso J, Marek K, Litvan I, Lang AE, Halliday G, Goetz CG, Gasser T, Dubois B, Chan P, Bloem BR, Adler CH, Deuschl G (2015) MDS clinical diagnostic criteria for Parkinson’s disease. Mov Disord 30(12):1591–1601. 10.1002/mds.26424 26474316 10.1002/mds.26424

[CR39] Singer W (2022) Recent advances in establishing fluid biomarkers for the diagnosis and differentiation of alpha-synucleinopathies – a mini review. Clin Auton Res 32:291–297. 10.1007/s10286-022-00882-1 35895157 10.1007/s10286-022-00882-1 PMC10101699

[CR40] Sletten DM, Suarez GA, Low PA, Mandrekar J, Singer W (2012) COMPASS 31: a refined and abbreviated composite autonomic symptom score. Mayo Clin Proc 87(12):1196–1201. 10.1016/j.mayocp.2012.10.013 23218087 10.1016/j.mayocp.2012.10.013 PMC3541923

[CR41] Stemplewitz B, Keserü M, Bittersohl D, Buhmann C, Skevas C, Richard G, Hassenstein A (2015) Scanning laser polarimetry and spectral domain optical coherence tomography for the detection of retinal changes in Parkinson’s disease. Acta Ophthalmol 93(8):e672–e677. 10.1111/aos.12764 26066643 10.1111/aos.12764

[CR42] Tewarie P, Balk L, Costello F, Green A, Martin R, Schippling S, Petzold A (2012) The OSCAR-IB consensus criteria for retinal OCT quality assessment. PLoS ONE 7(4):e34823. 10.1371/journal.pone.0034823 22536333 10.1371/journal.pone.0034823 PMC3334941

[CR43] Vieira BDM, Radford RA, Chung RS, Guillemin GJ, Pountney DL (2015) Neuroinflammation in multiple system atrophy: response to and cause of α-synuclein aggregation. Front Cell Neurosci 9:437. 10.3389/fncel.2015.00437 26778958 10.3389/fncel.2015.00437 PMC4700780

[CR44] Vujosevic S, Bini S, Torresin T, Berton M, Midena G, Parrozzani R, Martini F, Pucci P, Daniele AR, Cavarzeran F, Midena E (2017) Hyperreflective retinal spots in diabetic eyes with and without diabetic macular edema: B-scan and en-face spectral domain optical coherence tomography evaluation. Retina 37(6):1092–1103. 10.1097/IAE.0000000000001304 27668929 10.1097/IAE.0000000000001304

[CR45] Wagner SK, Romero-Bascones D, Cortina-Borja M, Williamson DJ, Struyven RR, Zhou Y, Patel S, Weil RS, Antoniades CA, Topol EJ, Korot E, Foster PJ, Balaskas K, Ayala U, Barrenechea M, Gabilondo I, Schapira AHV, Khawaja AP, Patel PJ, Rahi JS, Denniston AK, Petzold A, Keane PA, for UK Biobank Eye & Vision Consortium (2023) Retinal optical coherence tomography features associated with incident and prevalent Parkinson disease. Neurology 101(16):e1581–e1593. 10.1212/WNL.0000000000207727 37604659 10.1212/WNL.0000000000207727 PMC10585674

[CR46] Wenning GK, Tison F, Seppi K, Sampaio C, Diem A, Yekhlef F, Ghorayeb I, Ory F, Galitzky M, Scaravilli T, Bozi M, Colosimo C, Gilman S, Shults CW, Quinn NP, Rascol O, Poewe W, Multiple System Atrophy Study Group (2004) Development and validation of the unified multiple system atrophy rating scale (UMSARS). Mov Disord 19(12):1391–1402. 10.1002/mds.20255 15452868 10.1002/mds.20255

[CR47] Wenning GK, Stankovic I, Vignatelli L, Fanciulli A, Calandra-Buonaura G, Seppi K, Palma JA, Meissner WG, Krismer F, Berg D, Cortelli P, Freeman R, Halliday G, Höglinger G, Lang A, Ling H, Litvan I, Low P, Miki Y, Panicker J, Pellecchia MT, Quinn N, Sakakibara R, Stamelou M, Tolosa E, Tsuji S, Warner T, Poewe W, Kaufmann H (2022) The movement disorder society criteria for the diagnosis of multiple system atrophy. Mov Disord 37(6):1131–1148. 10.1002/mds.29005 35445419 10.1002/mds.29005 PMC9321158

[CR48] Zhang JR, Cao YL, Li K, Wang F, Wang YL, Wu JJ, Pei SF, Chen J, Mao CJ, Liu CF (2021) Correlations between retinal nerve fiber layer thickness and cognitive progression in Parkinson’s disease: a longitudinal study. Parkinsonism Relat Disord 82:92–97. 10.1016/j.parkreldis.2020.11.025 33271462 10.1016/j.parkreldis.2020.11.025

[CR49] Zhou WC, Tao JX, Li J (2021) Optical coherence tomography measurements as potential imaging biomarkers for Parkinson’s disease: a systematic review and meta-analysis. Eur J Neurol 28(3):763–774. 10.1111/ene.14613 33107159 10.1111/ene.14613

