# Supplementary material for: Optical coherence tomography reveals retinal structural abnormalities in α-synucleinopathies: insights from the Padua-CESNE cohort
Source: J Neural Transm (Vienna). 2025 Apr 15;132(7):1013–22. doi: 10.1007/s00702-025-02918-y (PMC12208970; doi:10.1007/s00702-025-02918-y)
Supplement: Supplementary file 1 — Supplementary file1 (DOCX 412 kb) [file 702_2025_2918_MOESM1_ESM.docx]

**Supplementary Materials**

Table 1. HRF counting in PD, MSA and healthy controls

|  | **PD** | | **MSA** | | **HC** | | **ANCOVA** |
| --- | --- | --- | --- | --- | --- | --- | --- |
|  | mean | SD | mean | SD | mean | SD | p-value |
| **HRF INL** | 2.1 | 0.3 | 6.1 | 0.5 | 2.5 | 0.1 | **<0.001** |
| **HRF GCIP** | 5.1 | 4.5 | 1.3 | 0.9 | 1.1 | 0.6 | **<0.001** |
| **HRF IRL** | 7.2 | 9.1 | 7.5 | 0.1 | 3.6 | 0.9 | **<0.001** |

Table 2. Skin biopsies results including IHC scores.

|  |  | **PD** | | **MSA** | |
| --- | --- | --- | --- | --- | --- |
|  |  |  |  |  |  |
| **Skin IHC score** | **0** | 4 | | 4 | |
|  | **0.25** | 5 | | 3 | |
|  | **0.50** | 3 | | 4 | |
|  | **0.75** | 1 | | 1 | |
|  | **1** | 1 | | 0 | |
|  | **Score (mean; SD)** | 0.32 | 0.30 | 0.27 | 0.27 |

Table 3. Anatomical localization of α-syn in skin biopsies of PD and MSA patients.

|  | **PD** | **MSA** |
| --- | --- | --- |
| **Anatomical localization of α-syn** |  |  |
| Vascular innervation | 9 | 4 |
| Glandular innervation | 4 | 4 |
| Free nerve endings | 1 | 1 |
| Hypodermal/dermal nerve bundles | 4 | 5 |

eFig 1. OCT mean thickness in PD and MSA population


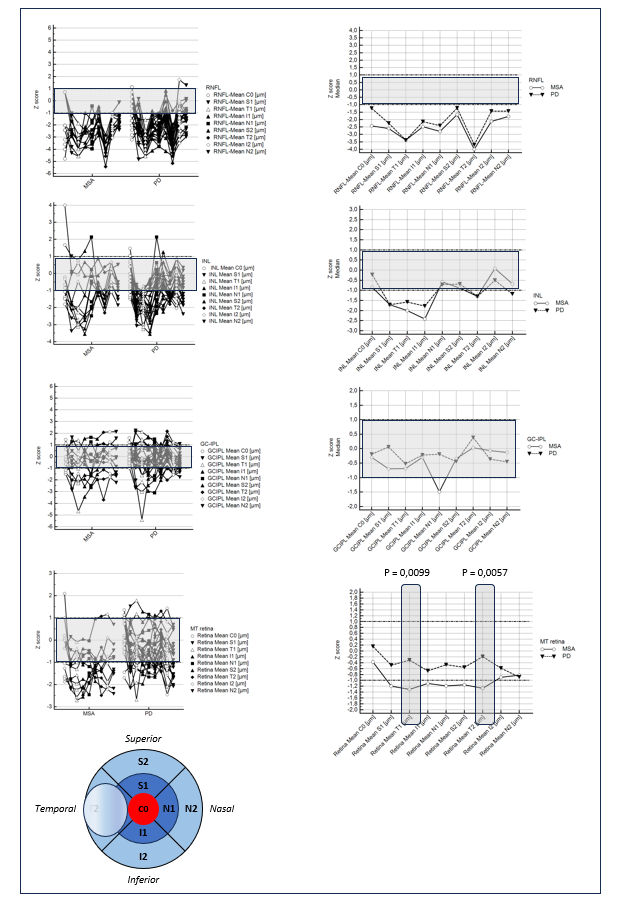


b

a

Legend. Z-scores from OCT central, inner, and outer temporal, nasal, inferior, and superior measures were compared between PD and MSA using Mann Whitney U test. A Bonferroni adjusted threshold p< 0.0055 was considered. a) A dot&line diagram was used to compare the single patient OCT profile in PD and MSA subgroups. B) median z scores values comparing MSA and PD profile. Normal range was outlined in the gray region included in -1 / +1 Z-score range.

eFig 2. Correlations between disease specific OCT-markers and clinical features


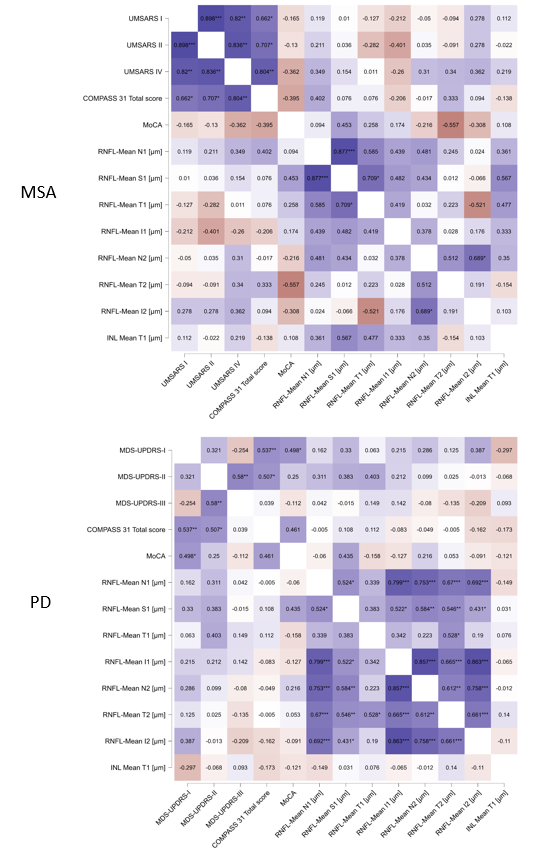


Legend. Partial Spearman's rho heatmap **c**onditioned on variable *age at visit; * p <0.05, ** p <0.01, *** p <0.001*
